# 2-(2,4,6-Trichloro­phen­oxy)ethyl bromide

**DOI:** 10.1107/S1600536809043256

**Published:** 2009-10-28

**Authors:** Jin-feng Yao, Wen-ge Yang, Xiao-lei Zhao, Lei Shen, Yong-hong Hu

**Affiliations:** a State Key Laboratory of Materials-Oriented Chemical Engineering, School of Pharmaceutical Sciences, Nanjing University of Technology, Xinmofan Road No. 5 Nanjing, Nanjing 210009, People’s Republic of China; b State Key Laboratory of Materials-Oriented Chemical Engineering, College of Life Science and Pharmaceutical Engineering, Nanjing University of Technology, Xinmofan Road No. 5 Nanjing, Nanjing 210009, People’s Republic of China

## Abstract

In the title compound, C_8_H_6_BrCl_3_O, there is a weak intra­molecular C—H⋯Cl hydrogen bond  involving the O bound methylene group. Intermolecular Cl⋯Cl contacts [3.482 (2) Å] are present in the crystal structure.

## Related literature

The title compound is used as an inter­mediate in the production of Prochloraz, a broad-spectrum imidazole fungicide widely used in gardening and agriculture. For the fungicidal properties of Prochloraz, see: Copping *et al.* (1984 ▶). For the preparation, see: Howard & Alfred (1982 ▶). For bond-length data, see: Allen *et al.* (1987 ▶).
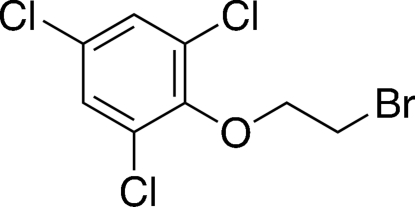


## Experimental

### 

#### Crystal data

C_8_H_6_BrCl_3_O
*M* *_r_* = 304.39
Triclinic, 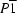

*a* = 4.0550 (8) Å
*b* = 8.6270 (17) Å
*c* = 15.183 (3) Å
α = 90.73 (3)°
β = 94.81 (3)°
γ = 90.42 (3)°
*V* = 529.21 (18) Å^3^
*Z* = 2
Mo *K*α radiation
μ = 4.60 mm^−1^
*T* = 293 K
0.20 × 0.10 × 0.10 mm

#### Data collection

Enraf–Nonius CAD-4 diffractometer
Absorption correction: ψ scan (North *et al.*, 1968 ▶) *T*_min_ = 0.460, *T*_max_ = 0.656
2215 measured reflections
1919 independent reflections
1280 reflections with *I* > 2σ(*I*)
*R*_int_ = 0.041
3 standard reflections every 200 reflections intensity decay: 1%

#### Refinement

*R*[*F* ^2^ > 2σ(*F* ^2^)] = 0.045
*wR*(*F* ^2^) = 0.123
*S* = 1.01
1919 reflections
118 parameters
H-atom parameters constrained
Δρ_max_ = 0.37 e Å^−3^
Δρ_min_ = −0.42 e Å^−3^

### 

Data collection: *CAD-4 Software* (Enraf–Nonius, 1989 ▶); cell refinement: *CAD-4 Software*; data reduction: *XCAD4* (Harms & Wocadlo, 1995 ▶); program(s) used to solve structure: *SHELXS97* (Sheldrick, 2008 ▶); program(s) used to refine structure: *SHELXL97* (Sheldrick, 2008 ▶); molecular graphics: *SHELXTL* (Sheldrick, 2008 ▶); software used to prepare material for publication: *PLATON* (Spek, 2009 ▶).

## Supplementary Material

Crystal structure: contains datablocks global, I. DOI: 10.1107/S1600536809043256/pv2217sup1.cif

Structure factors: contains datablocks I. DOI: 10.1107/S1600536809043256/pv2217Isup2.hkl

Additional supplementary materials:  crystallographic information; 3D view; checkCIF report

## Figures and Tables

**Table 1:** Hydrogen-bond geometry (Å, °)

*D*—H⋯*A*	*D*—H	H⋯*A*	*D*⋯*A*	*D*—H⋯*A*
C2—H2*A*⋯Cl3	0.97	2.81	3.276 (6)	110

**Fig. 1.**
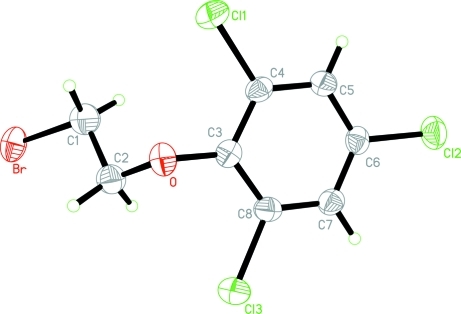
The molecular structure of the title compound with the atom numbering scheme. Displacement ellipsoids are drawn at 30% probability levels.

**Fig. 2.**
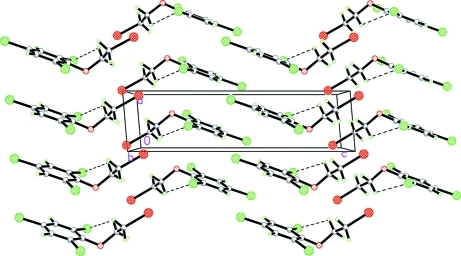
A packing diagram of the title compound. Intramolecular hydron bonds are shown as dashed lines.

## supplementary crystallographic information

### Comment

Prochloraz, *N*-propyl-*N*-[2-(2,4,6-trichlorophenoxy)-ethyl] -1*H*-imidazole-1-carboxamide, is a broad-spectrum imidazole fungicide (Copping *et al.*, 1984). As part of our studies in the synthesis of Prochloraz, the title compound (I), which is used as the key intermediate, has been synthesized. We report herein the crystal structure of the title compound.

In the molecule of the title compound (Fig. 1), the bond lengths and angles are within normal ranges (Allen *et al.*, 1987). In the crystal structure, intramolecular C—H···Cl interactions (Table 1) may be effective in the stabilization of the structure.

### Experimental

The title compound was prepared by following a reported procedure (Howard & Alfred, 1982). 2,4,6-Trichlorophenol (15.8 g ) and sodium hydroxide (4.8 g) were dissolved in 28 ml water and added dropwise to an excess of ethylene dibromide (75.6 g). The reaction mixture was heated under reflux for ten hours. The residue was extracted with 3 *x* 20 ml dichlormethane, and then methylene chloride phase was washed with water, dried and evaporated to dryness under reduced pressure. Fractionation under reduced pressure yielded the title compound as a colorless oil whaich was then cooled to give 18.1 g white solid (75.2%). Crystals of (I) suitable for X-ray diffraction were obtained by slow evaporation of an ethanol solution of (I).

### Refinement

H atoms were positioned geometrically with C—H = 0.93 and 0.97 Å for aromatic and methylene H atoms, respectively, and constrained to ride on their parent atoms, with *U*_iso_(H) = 1.2 times *U*_eq_(C).

### Crystal data

**Table:**

C_8_H_6_BrCl_3_O	*Z* = 2
*M**_r_* = 304.39	*F*(000) = 296
Triclinic, *P*1	*D*_x_ = 1.910 Mg m^−^^3^
Hall symbol: -P 1	Mo *K*α radiation, λ = 0.71073 Å
*a* = 4.0550 (8) Å	Cell parameters from 25 reflections
*b* = 8.6270 (17) Å	θ = 10–14°
*c* = 15.183 (3) Å	µ = 4.60 mm^−^^1^
α = 90.73 (3)°	*T* = 293 K
β = 94.81 (3)°	Block, colorless
γ = 90.42 (3)°	0.20 × 0.10 × 0.10 mm
*V* = 529.21 (18) Å^3^	

### Data collection

**Table:**

Enraf–Nonius CAD-4 diffractometer	1280 reflections with *I* > 2σ(*I*)
Radiation source: fine-focus sealed tube	*R*_int_ = 0.041
graphite	θ_max_ = 25.3°, θ_min_ = 1.4°
ω/2θ scans	*h* = 0→4
Absorption correction: ψ scan (North *et al.*, 1968)	*k* = −10→10
*T*_min_ = 0.460, *T*_max_ = 0.656	*l* = −18→18
2215 measured reflections	3 standard reflections every 200 reflections
1919 independent reflections	intensity decay: 1%

### Refinement

**Table:**

Refinement on *F*^2^	Primary atom site location: structure-invariant direct methods
Least-squares matrix: full	Secondary atom site location: difference Fourier map
*R*[*F*^2^ > 2σ(*F*^2^)] = 0.045	Hydrogen site location: inferred from neighbouring sites
*wR*(*F*^2^) = 0.123	H-atom parameters constrained
*S* = 1.01	*w* = 1/[σ^2^(*F*_o_^2^) + (0.066*P*)^2^] where *P* = (*F*_o_^2^ + 2*F*_c_^2^)/3
1919 reflections	(Δ/σ)_max_ < 0.001
118 parameters	Δρ_max_ = 0.37 e Å^−^^3^
0 restraints	Δρ_min_ = −0.42 e Å^−^^3^

### Special details

**Table:**

Geometry. All e.s.d.'s (except the e.s.d. in the dihedral angle between two l.s. planes) are estimated using the full covariance matrix. The cell e.s.d.'s are taken into account individually in the estimation of e.s.d.'s in distances, angles and torsion angles; correlations between e.s.d.'s in cell parameters are only used when they are defined by crystal symmetry. An approximate (isotropic) treatment of cell e.s.d.'s is used for estimating e.s.d.'s involving l.s. planes.
Refinement. Refinement of *F*^2^ against ALL reflections. The weighted *R*-factor *wR* and goodness of fit *S* are based on *F*^2^, conventional *R*-factors *R* are based on *F*, with *F* set to zero for negative *F*^2^. The threshold expression of *F*^2^ > σ(*F*^2^) is used only for calculating *R*-factors(gt) *etc*. and is not relevant to the choice of reflections for refinement. *R*-factors based on *F*^2^ are statistically about twice as large as those based on *F*, and *R*- factors based on ALL data will be even larger.

### Fractional atomic coordinates and isotropic or equivalent isotropic displacement parameters (Å^2^)

**Table:**

	*x*	*y*	*z*	*U*_iso_*/*U*_eq_	
Br	0.06298 (16)	0.24181 (8)	−0.05453 (4)	0.0696 (3)	
O	0.6382 (9)	0.3089 (4)	0.1806 (2)	0.0537 (9)	
Cl1	0.6363 (4)	−0.01264 (17)	0.24134 (11)	0.0689 (5)	
Cl2	0.1372 (4)	0.22953 (19)	0.52835 (10)	0.0707 (5)	
Cl3	0.4167 (5)	0.59982 (17)	0.26392 (11)	0.0777 (5)	
C1	0.3028 (15)	0.1966 (7)	0.0606 (4)	0.0651 (16)	
H1A	0.1585	0.1417	0.0977	0.078*	
H1B	0.4922	0.1318	0.0521	0.078*	
C2	0.4125 (15)	0.3430 (7)	0.1030 (4)	0.0609 (15)	
H2A	0.2235	0.4002	0.1208	0.073*	
H2B	0.5249	0.4058	0.0620	0.073*	
C3	0.5043 (12)	0.2915 (6)	0.2586 (3)	0.0436 (12)	
C4	0.4945 (12)	0.1458 (6)	0.2977 (4)	0.0467 (13)	
C5	0.3811 (13)	0.1253 (6)	0.3795 (3)	0.0486 (13)	
H5A	0.3760	0.0273	0.4042	0.058*	
C6	0.2752 (13)	0.2530 (6)	0.4241 (3)	0.0476 (13)	
C7	0.2808 (13)	0.3971 (6)	0.3887 (4)	0.0506 (14)	
H7A	0.2071	0.4822	0.4195	0.061*	
C8	0.3966 (13)	0.4152 (6)	0.3068 (4)	0.0482 (13)	

### Atomic displacement parameters (Å^2^)

**Table:**

	*U*^11^	*U*^22^	*U*^33^	*U*^12^	*U*^13^	*U*^23^
Br	0.0586 (4)	0.0971 (5)	0.0522 (4)	0.0021 (3)	0.0018 (3)	−0.0099 (3)
O	0.045 (2)	0.070 (2)	0.046 (2)	0.0014 (18)	0.0040 (19)	0.0043 (18)
Cl1	0.0775 (11)	0.0580 (9)	0.0722 (10)	0.0176 (8)	0.0128 (9)	−0.0101 (7)
Cl2	0.0801 (11)	0.0855 (11)	0.0482 (9)	0.0115 (9)	0.0131 (8)	0.0051 (8)
Cl3	0.1140 (14)	0.0486 (8)	0.0695 (11)	−0.0029 (9)	0.0020 (10)	0.0059 (7)
C1	0.053 (4)	0.069 (4)	0.075 (4)	−0.002 (3)	0.018 (3)	0.002 (3)
C2	0.062 (4)	0.058 (3)	0.064 (4)	−0.001 (3)	0.016 (3)	0.004 (3)
C3	0.033 (3)	0.052 (3)	0.045 (3)	0.001 (2)	−0.001 (2)	−0.003 (2)
C4	0.044 (3)	0.045 (3)	0.051 (3)	0.008 (2)	−0.001 (3)	−0.007 (2)
C5	0.049 (3)	0.046 (3)	0.051 (3)	0.004 (2)	0.003 (3)	0.003 (3)
C6	0.041 (3)	0.059 (3)	0.042 (3)	0.005 (3)	−0.005 (2)	−0.001 (3)
C7	0.052 (3)	0.050 (3)	0.050 (3)	0.009 (3)	−0.002 (3)	−0.004 (3)
C8	0.048 (3)	0.041 (3)	0.054 (3)	0.001 (2)	−0.003 (3)	0.001 (2)

### Geometric parameters (Å, °)

**Table:**

Br—C1	1.973 (6)	C2—H2B	0.9700
O—C3	1.353 (6)	C3—C8	1.380 (7)
O—C2	1.464 (7)	C3—C4	1.398 (7)
Cl1—C4	1.731 (5)	C4—C5	1.373 (7)
Cl2—C6	1.737 (5)	C5—C6	1.376 (7)
Cl3—C8	1.733 (5)	C5—H5A	0.9300
C1—C2	1.459 (8)	C6—C7	1.361 (7)
C1—H1A	0.9700	C7—C8	1.375 (8)
C1—H1B	0.9700	C7—H7A	0.9300
C2—H2A	0.9700		
			
C3—O—C2	117.4 (4)	C5—C4—C3	122.1 (5)
C2—C1—Br	108.5 (4)	C5—C4—Cl1	119.2 (4)
C2—C1—H1A	110.0	C3—C4—Cl1	118.6 (4)
Br—C1—H1A	110.0	C4—C5—C6	118.5 (5)
C2—C1—H1B	110.0	C4—C5—H5A	120.8
Br—C1—H1B	110.0	C6—C5—H5A	120.8
H1A—C1—H1B	108.4	C7—C6—C5	121.4 (5)
C1—C2—O	108.5 (5)	C7—C6—Cl2	119.5 (4)
C1—C2—H2A	110.0	C5—C6—Cl2	119.1 (4)
O—C2—H2A	110.0	C6—C7—C8	119.2 (5)
C1—C2—H2B	110.0	C6—C7—H7A	120.4
O—C2—H2B	110.0	C8—C7—H7A	120.4
H2A—C2—H2B	108.4	C7—C8—C3	122.1 (5)
O—C3—C8	122.7 (5)	C7—C8—Cl3	118.9 (4)
O—C3—C4	120.4 (4)	C3—C8—Cl3	118.9 (4)
C8—C3—C4	116.7 (5)		
			
Br—C1—C2—O	−170.3 (3)	C4—C5—C6—C7	0.1 (8)
C3—O—C2—C1	−90.6 (6)	C4—C5—C6—Cl2	−179.0 (4)
C2—O—C3—C8	−75.1 (6)	C5—C6—C7—C8	−0.4 (8)
C2—O—C3—C4	110.3 (5)	Cl2—C6—C7—C8	178.7 (4)
O—C3—C4—C5	175.5 (5)	C6—C7—C8—C3	0.8 (8)
C8—C3—C4—C5	0.6 (7)	C6—C7—C8—Cl3	−177.7 (4)
O—C3—C4—Cl1	−3.4 (6)	O—C3—C8—C7	−175.7 (5)
C8—C3—C4—Cl1	−178.4 (4)	C4—C3—C8—C7	−0.9 (8)
C3—C4—C5—C6	−0.2 (8)	O—C3—C8—Cl3	2.8 (7)
Cl1—C4—C5—C6	178.8 (4)	C4—C3—C8—Cl3	177.7 (4)

### Hydrogen-bond geometry (Å, °)

**Table:**

*D*—H···*A*	*D*—H	H···*A*	*D*···*A*	*D*—H···*A*
C2—H2A···Cl3	0.97	2.81	3.276 (6)	110
